# The Ex-Soldier

**Published:** 1920-09-25

**Authors:** 


THE PUBLIC WELL-BEING—(*continued*)

### The Ex-Soldier.

The comments made in The Hospital on Lord Haig's appeal on behalf of the ex-soldiers have been quoted with appreciation in several newspapers. We are glad to know that public interest is at last being aroused in a matter which so closely concerns the public honour. Since the appearance of our articles on this subject reports have been sent to us of meetings of boards of guardians in all parts of the country at which scathing remarks were made on the scandal of ex-Service men having to go to the Poor Law for relief. Fortunately, the majority of people have at last discarded the view that, because the comparatively few criminals and vagabonds who joined the forces during the war have remained criminals and vagabonds after the war, the demobilised soldier, as a whole, must be judged from the lowest standard of morality and the "will to work." We need offer no apology for continuing to press on the community the claim of the still unemployed soldiers.

